# Influence of Time of Mission on Correct Diagnosis by the Prehospital Emergency Physician: A Retrospective Study

**DOI:** 10.1155/2019/3727081

**Published:** 2019-04-02

**Authors:** Nikolai Ramadanov, Roman Klein, Nevena Ramadanova, Wilhelm Behringer

**Affiliations:** ^1^Zentrum für Notfallmedizin, Universitätsklinikum Jena, Friedrich-Schiller-Universität Am Klinikum 1, 07747 Jena, Germany; ^2^Klinik für Unfall- und Wiederherstellungschirurgie, Klinikum Ernst von Bergmann Potsdam, Charlottenstraße. 72, 14467 Potsdam, Germany; ^3^Orthopädie, Unfallchirurgie und Sporttraumatologie, Marienhaus Klinikum Hetzelstift, Stiftstraße 10, 67434 Neustadt, Germany; ^4^Innere Medizin, Klinik Ernst v. Bergmann Bad Belzig, Niemegker Str. 45, 14806 Bad Belzig, Germany

## Abstract

**Objectives:**

The objective of this retrospective study was to examine the diagnostic matching (DM) between the prehospital diagnosis by the prehospital emergency physicians and the hospital discharge diagnosis, adjusted for time of mission.

**Methods:**

Over a period of 12 months, all patient care reports of the emergency medical services in Bad Belzig were examined. By systematically comparing the prehospital suspected diagnosis to the discharge diagnosis, the DM was determined after careful examination of the entire course of each patient's case, blinded to time of mission. The results were tested for statistically significant results using the Chi-square test for nominal data and the Mann-Whitney U test for nonnormally distributed independent samples.

**Results:**

The DM occurred in 52%, it occurred partially in 24%, and it did not occur in 24% of 580 included cases. The DM showed clear fluctuation over 24 hours, with the worst results at 4 and 5 a.m. and the best results at 6 a.m. and 3 p.m.

**Conclusions:**

The DM appears to depend directly on the time of mission. Decreased performance and concentration at night might be a cause for incorrect diagnoses by prehospital emergency physician in the early morning hours. Future studies need to investigate the effect of different shift planning on performance.

## 1. Introduction

Prehospital emergency physicians often have to perform missions with critically ill patients at night, when the circadian rhythm of the human being calls for a recovery phase. In addition to the generally high level of workload that doctors experience, emergency physicians are the most frequently confronted to extreme situations of all occupational groups [1]. Numerous studies have shown the negative impact of sleep deprivation on night-shift physicians. Even in a benign work schedule, the effects of sleep loss on attention and working memory in resident anaesthetists after night duty are obvious [2]. Cognitive performance of physicians decreases significantly after a night shift [3–5]. A night shift on call even has negative effects on the mood of the physicians [4, 5]. Physicians on overnight call and post-call are more likely to commit medication-ordering errors [6]. These facts also have an influence on the patient satisfaction. Patients seen by their general practitioner before and after a night on call were less satisfied than patients seen before and after a night off duty [7]. In general, shift work at night has negative effects on sleep, performance, accident risk, and health outcomes [8]. Very interesting is the finding that partial sleep deprivation has an even stronger negative influence on human functioning than either long-term or short-term sleep deprivation [9]. Few studies have been done so far on the diagnostic matching (DM) between prehospital diagnoses by the prehospital emergency physician and hospital discharge diagnoses [10–12]. None of those studies examined sufficiently the influence of time of mission on DM. This topic should be examined closely, because there is an association between incorrect admission diagnoses increased mortality and morbidity [10]. The objective of this retrospective study was to examine the DM between prehospital diagnoses by the prehospital emergency physician and the hospital discharge diagnoses, adjusted for time of mission.

## 2. Methods

### 2.1. Description of the Local EMS System

The local emergency medical services (EMS) system in Bad Belzig worked according to the typical for Germany rendez-vous principle. The prehospital emergency physician, accompanied by a paramedic with a vehicle on duty, had to meet the ambulance vehicle with other paramedics at the patient location. The prehospital emergency physicians responded just to a selected cohort of critical patient cases (2,7 cases/day). The paramedics responded to the rest of emergency calls. The shift duration of the prehospital emergency physician was 24 hours, starting at 7:30 a.m. Within the regular working hours in the hospital, from 7:30 a.m. to 4 p.m., the emergency physicians were simultaneously burdened with clinic internal work and with their emergency missions. The documentation of diagnoses and mission details were recorded in prehospital emergency physician patient care report. The EMS point in Bad Belzig has wide distance to the hospitals, up to one hour to Potsdam. 6 of 13 prehospital emergency physicians had the higher qualification “Zusatzbezeichnung Notfallmedizin” and 7 of them the lower qualification “Fachkunde Rettungsdienst.” Table 1 shows further characteristics of the prehospital emergency physicians.

### 2.2. Data Collection

All prehospital emergency physicians' patient care reports (DIVI protocol 4.2) of the EMS in Bad Belzig in the period from July 1, 2013, to June 30, 2014, were examined. The time of mission was recorded rounded up to an entire hour. The discharge diagnoses were taken from the hospital information system in Bad Belzig (SAP version 7400.1.0.1093). Further discharge summaries were included from neighbouring hospitals (Klinikum Ernst von Bergmann Potsdam, Asklepios Fachklinik Brandenburg, Städtisches Krankenhaus Brandenburg, and Johanniter Krankenhaus in Fläming Treuenbrietzen). The study was approved by the ethical committee of the University of Jena (No. 4522-08/15/15).

### 2.3. Diagnostic Matching

By systematically comparing the prehospital suspected diagnosis to the discharge diagnosis, the DM was determined blinded to time of mission by the consensus of three physicians after careful examination of the entire course of each patient's case. During the determination process the names of the patients and the prehospital emergency physician also are blinded. All diagnoses from the patient care report and only mission-related discharge diagnoses were rated. Complications that occurred during hospital stay were not rated. The DM occurred when every prehospital diagnosis could be confirmed among the diagnoses of the discharge summary and none of the prehospital diagnoses were missing. The DM occurred partially when only a part of the prehospital diagnoses could be confirmed among the diagnoses of the discharge summary. The DM did not occur when not even one prehospital diagnosis could be confirmed among the diagnoses of the discharge summary.

### 2.4. Statistics

The statistical calculations were performed using IBM SPSS Statistics 19 for Windows. The determined DM was tested for relevant differences, depending on the time-of-mission factor. The hours with the best and worst results for DM were summarised and compared with the remaining hours. Nominal data were compared using the Chi-square test, and the nonnormally distributed independent samples were compared by using the Mann-Whitney U test. Statistical significance was assumed when P < 0.05.

## 3. Results

The DM occurred in 52% (303 cases), it occurred partially in 24% (139 cases), and it did not occur in 24% (138 cases) of the 580 included cases.

### 3.1. Exclusion of Cases

**Figure 1:**
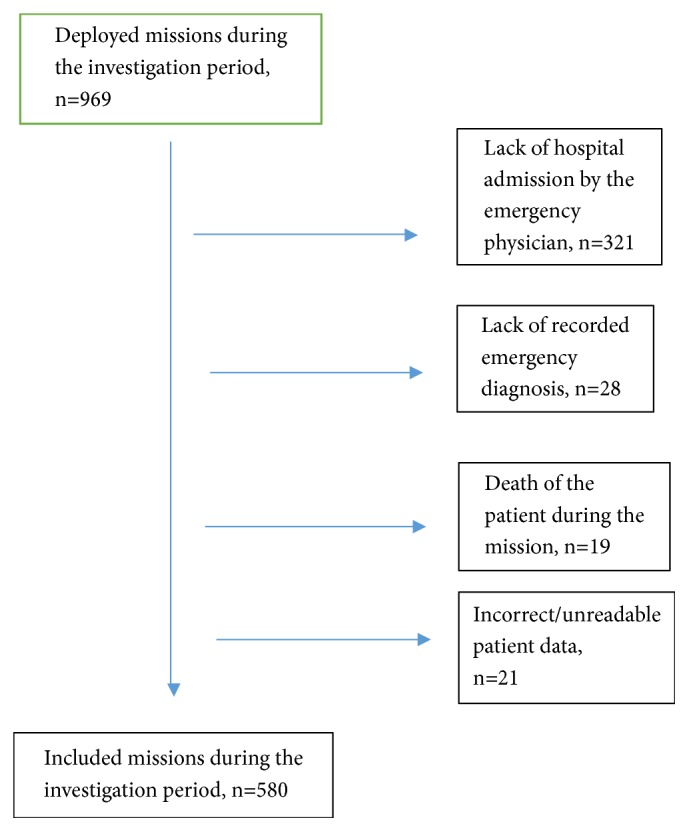
Inclusion chart.

389 patient care reports were excluded from the study for the following reasons: lack of hospital admission by the emergency physician, lack of recorded emergency diagnosis, death of the patient during the mission, or incorrect/unreadable patient data (see Figure 1).

### 3.2. Spectrum of Discharge Diagnoses

A total of 747 hospital discharge diagnoses after admission by the prehospital emergency physician were made for the 580 included cases. In 126 cases, 2 discharge diagnoses were made for one patient, in 19 cases, 3 discharge diagnoses, and in 1 case, 4 discharge diagnoses.  Table 2 shows the 10 most common discharge diagnoses after admission by the prehospital emergency physician.  Table 3 shows the distribution of the most common discharge diagnoses, summarized by specialty.

### 3.3. Time of Mission Factor

#### 3.3.1. Diagnostic Matching over 24 Hours

**Figure 2:**
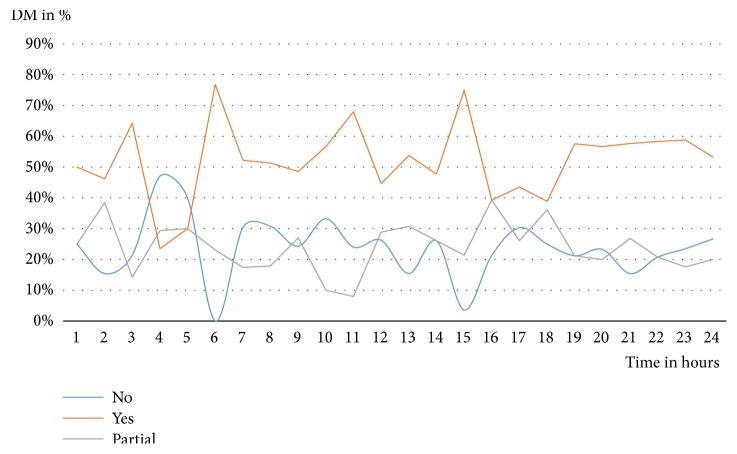
Fluctuations of the DM over 24 hours in %.

Figure 2 shows the DM between prehospital diagnoses by prehospital emergency physicians and the hospital discharge diagnoses over 24 hours. The proportion of the occurring DM and the missing DM showed a mirror-inverted variation over the course of the day with opposite peak values around 4 and 5 a.m. as well as at 6 a.m. and 3 p.m. The proportion of partial DM varied over 24 hours between 9% and 40%.

#### 3.3.2. DM between the Mission Times with the Worst and Best Results for DM and the Rest of the Daytime

The comparison of DM between the times of mission with the worst (at 4 and 5 a.m.) and best (at 6 a.m. and 3 p.m.) results for DM and the rest of the daytime showed statistically significant results (p = 0.001).

### 3.4. Duration of Mission

The duration of the mission was defined as the time interval between the arrival of the prehospital emergency physician at the patient and the departure from the place of his mission. The median nocturnal time of mission was statistically significant, longer than the rest of the times of mission, 27 (IQR 16–31) minutes versus 20 (IQR 14–-25) minutes (p = 0.012).

## 4. Discussion

The correctness of the suspected diagnosis was estimated retrospectively by calculating the DM in the present study. The DM occurred in 52%, it occurred partially in 24%, and it did not occur in 24% of the 580 included cases. Our investigation showed further clear fluctuation in DM over 24 hours with high matching at 6 a.m. and 3 p.m. and low matching at 4 a.m. and 5 a.m. Over 24 hours, this resulted in a regular, mirror-inverted curve between the correct and incorrect diagnoses by the prehospital emergency physician.

The highest part of incorrect diagnoses occurred at the nonphysiological working time at night. We suppose that the shortage of sleep and the lack of recovery after a prolonged working day (24 hours) might have led to a significant decrease in concentration of the prehospital emergency physician. The highest part of correct diagnoses, however, occurred at 6 a.m. and 3 p.m. The explanation for this could be the 24-hour shift work at the emergency medical services point in Bad Belzig. During the investigation period from July 1, 2013, to June 30, 2014, the change of the physician's shift was at 7:30 a.m. Within the regular working hours in the hospital, from 7:30 a.m. to 4 p.m., the emergency physicians were simultaneously burdened with clinic internal work and with the prehospital emergency missions. From 4 p.m. onward, the prehospital emergency physicians were only responsible for their missions. The times of mission with the most correct diagnoses were in both cases times shortly before a serious decrease in the working load, so an emotional factor on the part of the emergency physician may have influenced the DM here.

By comparing the duration of missions during the problematic nocturnal time of mission with the rest of the daytime, we showed that the prehospital emergency physician did not act according to a superficial mission strategy (load and go). On the contrary, a delayed treatment period at the problematic nocturnal mission time was determined, which we suppose might be explained by the fatigue of the prehospital emergency physician.

### 4.1. Discussion of the Results Compared to the Literature

The correctness of the prehospital diagnoses in general was compared to the results of these emergency medical services. In the study by Arntz [11] about two-thirds of the 2254 included cases were carried out by internist prehospital emergency physicians, as in Bad Belzig. However, in only 10% of cases the suspected prehospital diagnosis was incorrect [11]. In the study by Heuer with 355 ambulance and 241 helicopter cases in only 9,9% of cases the suspected prehospital diagnosis was incorrect [13]. When comparing those studies directly, the different methods used to evaluate the DM must be borne in mind.

Numerous studies in various tests have already shown a reduction of mood, concentration, and performance in sleep-deficient physicians and shift workers [1–9]. In general, these results are consistent with ours, although most of those studies examined the condition of shift workers after night shifts. In our study, the negative effects are already visible in the night of the 24-hour duty. The study by Peter also compared the suspected diagnoses of the ambulance emergency physicians with the hospital discharge diagnoses and examined the time-of-mission factor [12]. On the basis of 2,400 included emergency missions, there were not statistically significant results toward more misdiagnosis during the night (00:00–07:00) compared to the rest of the time (07:00–00:00) (35% vs. 22%, p = 0.78).

### 4.2. Possible Implications

The accumulation of faulty suspicious diagnoses in the night hours could lead to fatal consequences for patient outcome. Possible countermeasures to counteract the negative effects of night service by shift workers have already been discussed in detail [8]. However, a more effective solution than the reduction of night services could not be found. Conditions should be set up to prevent prehospital emergency physicians from becoming exhausted and to provide for them an adequate level of motivation for adequate patient care. Because the ambulance emergency physician's missions often have an acute life-threatening character, no compromises should be allowed regarding the workload of these physicians. By establishing regular tests to monitor the exhaustion of ambulance emergency physicians, potential problems could be solved in time and the physician's service system and plan could be adjusted according to the results of the tests. The double burden on the prehospital emergency physician of ambulance missions and internal hospital tasks should be avoided. An accumulation of sleep deficit over several services is quite possible and can be avoided by checking the permissible service frequency. In a prospective analysis, the results should be investigated to establish whether performance can be improved by omitting the double exposure due to internal hospital work or by instituting a 12-hour service.

### 4.3. Limitations to the Study

It must be considered that the present study is a retrospective analysis and thus it can only identify associations. There is a certain degree of subjectivity in the determination of dA in this study as well as in other studies, since the choice of different methods may lead to deviations in the results. The significance of findings is reduced by the lower volume of cases requiring transfer during night hours. Further studies are necessary to confirm the results. This study was carried out at an EMS location with a 24-hour shift, a mission frequency of 2.7 missions per day, and a significant additional burden on emergency physicians with internal hospital tasks. Since there are differences in the EMS system over the whole country, the described EMS location is not sufficiently representative. A geographically varying distribution of the diagnoses is conceivable. Transferring the results to other EMS locations and regions is therefore difficult. The different conditions at the EMS points might have an impact on the results of DM. Furthermore it is important to remember that throughout the entire course from mission to dismissal of each patient case there might be other factors which could potentially affect results.

## 5. Conclusion

In our study of 24-hour prehospital emergency physicians' services and the emergency department's attendance at regular working times by the same physician, 24% of prehospital emergency diagnoses did not correspond to hospital discharge diagnoses. The most common incorrect diagnoses were made in the night between 4 and 5 a.m. An increase of correct diagnoses was determined at daytime shortly before a serious decrease in the working load. The correct medical diagnosis appears to depend directly on the time of mission and thus to be subject to significant fluctuation in DM over 24 hours.

Future studies should examine whether the results can be improved by omitting double exposure to internal hospital work or by instituting a 12-hour service.

## Figures and Tables

**Table 1:** Listing of the mission distribution according to specialty and consultant/specialist status of the prehospital emergency physician.

Specialty and consultant/specialist status of the prehospital emergency physician	Number of missions by specialists (*n*)	Number of missions by consultants (*n*)	Total number of missions (n)
Internal medicine	100	262	362
Surgery	38	116	154
Anaesthesia	0	11	11
General practice	53	0	53

**Table 2:** 10 most common discharge diagnoses (n=747).

ICD	Designation	%
I10.91	hypertensive crisis	10,0
R55	syncope	6,0
I50.9	cardiac decompensation	5,5
I48.9	tachyarrhythmia absoluta/atrial fibrillation	4,3
E86	dehydration	3,9
I21.4/I21.3/I21.9	NSTEMI/STEMI/acute myocardial infarction	3,9
J15.9	pneumonia	3,7
I20.9	angina pectoris	3,3
J44.09	exacerbated COPD	3,1
R07.4	chest pain	2,4

**Table 3:** Distribution of the most common discharge diagnoses, summarized by specialty.

Designation	In percent (%)
Cardiology	32,2
Orthopedics and trauma surgery	10,7
Pulmonology	9,2
Neurology	7,7
Endocrinology	7,6

## Data Availability

The data used to support the findings of this study are available from the corresponding author upon request.

## Abbreviations

DM: Diagnostic matching
DIVI: Deutschte Interdisziplinäre Vereinigung für Intensiv- und Notfallmedizin
EMS: Emergency medical services
SAP: Systeme, Anwendungen und Produkte in der Datenverarbeitung
IBM SPSS: International Business Machines Corporation Statistical Package for the Social Sciences
IQR: Interquartile range.
